# Synthetic CT generation for MRI-guided adaptive radiotherapy in prostate cancer

**DOI:** 10.3389/fonc.2022.969463

**Published:** 2022-09-23

**Authors:** Shu-Hui Hsu, Zhaohui Han, Jonathan E. Leeman, Yue-Houng Hu, Raymond H. Mak, Atchar Sudhyadhom

**Affiliations:** Department of Radiation Oncology, Brigham and Women’s Hospital and Dana-Farber Cancer Institute, Boston, MA, United States

**Keywords:** MRI-guided therapy, adaptive radiotherapy, synthetic CT, deep learning, prostate radiotherapy

## Abstract

Current MRI-guided adaptive radiotherapy (MRgART) workflows require fraction-specific electron and/or mass density maps, which are created by deformable image registration (DIR) between the simulation CT images and daily MR images. Manual density overrides may also be needed where DIR-produced results are inaccurate. This approach slows the adaptive radiotherapy workflow and introduces additional dosimetric uncertainties, especially in the presence of the magnetic field. This study investigated a method based on a conditional generative adversarial network (cGAN) with a multi-planar method to generate synthetic CT images from low-field MR images to improve efficiency in MRgART workflows for prostate cancer. Fifty-seven male patients, who received MRI-guided radiation therapy to the pelvis using the ViewRay MRIdian Linac, were selected. Forty-five cases were randomly assigned to the training cohort with the remaining twelve cases assigned to the validation/testing cohort. All patient datasets had a semi-paired DIR-deformed CT-sim image and 0.35T MR image acquired using a true fast imaging with steady-state precession (TrueFISP) sequence. Synthetic CT images were compared with deformed CT images to evaluate image quality and dosimetric accuracy. To evaluate the dosimetric accuracy of this method, clinical plans were recalculated on synthetic CT images in the MRIdian treatment planning system. Dose volume histograms for planning target volumes (PTVs) and organs-at-risk (OARs) and dose distributions using gamma analyses were evaluated. The mean-absolute-errors (MAEs) in CT numbers were 30.1 ± 4.2 HU, 19.6 ± 2.3 HU and 158.5 ± 26.0 HU for the whole pelvis, soft tissue, and bone, respectively. The peak signal-to-noise ratio was 35.2 ± 1.7 and the structural index similarity measure was 0.9758 ± 0.0035. The dosimetric difference was on average less than 1% for all PTV and OAR metrics. Plans showed good agreement with gamma pass rates of 99% and 99.9% for 1%/1 mm and 2%/2 mm, respectively. Our study demonstrates the potential of using synthetic CT images created with a multi-planar cGAN method from 0.35T MRI TrueFISP images for the MRgART treatment of prostate radiotherapy. Future work will validate the method in a large cohort of patients and investigate the limitations of the method in the adaptive workflow.

## 1 Introduction

Online prostate adaptive radiotherapy allows for the generation of an optimal treatment plan based on daily changes to anatomy and has the potential to improve target coverage and reduce the toxicity to the surrounding organs such as rectum, bladder and urethra. Recently, the MR-linac treatment modality offers on-board MR images with high soft tissue contrast for improved tumor and normal tissue delineation compared to standard cone-beam CT based radiotherapy (1). Additionally, the system can deliver gated treatment by monitoring the prostate or critical organ positions to ensure high-precision treatment. Therefore, MRI-guided adaptive radiotherapy (MRgART) can potentially improve treatment outcomes by reducing inter-fraction and intra-fraction uncertainties in prostate treatments (2–4).

Current clinical procedures in MRgART require CT image acquisitions for dose calculations in treatment planning because of the lack of electron/mass density information on MR images. For each fraction, daily MR images are acquired and deformably registered to the original simulation MR image, calculating a daily deformation vector field (DVF). The deformable image registration (DIR) DVF is then applied to original simulation CT images to propagate the electron/mass density information in the coordinate frame of daily MR images. Any remaining differences in the body contour and air pockets between the two image sets are compensated for by means of user-driven override structures with densities equal to soft tissue and air. The process of DIR and density overrides must be repeated for each fraction in the adaptive radiotherapy workflow. This approach is labor intensive, subject to user bias and interpretation, slows the adaptive radiotherapy workflow and introduces additional dosimetric uncertainties, especially in the presence of the magnetic field (5, 6). Thus, generating synthetic CT from daily MRI images would streamline the MRgART workflow and reduce uncertainties caused by image registration and manual density overrides.

Challenges in generating synthetic CT from MRI include the lack of correlation between the MRI intensity and tissue electron density, the dependence of the MRI contrast on scanning parameters, and the difficulty in distinguishing air from bone on MR images as both air and dense bone have little to no signal on MRI. Several methods have been proposed for generating synthetic CT images from MR images in various body sites and have been reviewed comprehensively in prior studies (7–11). These methods included bulk density assignment, atlas-based methods, voxel-based methods and machine learning methods. Most studies focused on MR simulators (1.5T and 3T). Additionally, these methods may require special scanning sequences or multiple pulse sequences, the most common being Dixon and zero echo time (ZTE)/ultrashort TE (UTE). Few studies have reported synthetic CT methods for low-field MRgART, such as ViewRay MRIdian (0.35T) (12–14). Generating synthetic CT images from low-field MR images can be more challenging due to the lower signal-to-noise ratio compared to higher field strength and limited selection of scanning protocols (usually using a true fast imaging with steady-state precession (TrueFISP) sequence for fast and low-noise imaging at low-field MRI). Therefore, tissue classification *via* tissue-contrast optimized scanning protocols may not be feasible for low-field MRgART because of the need to compromise with fast imaging, particularly for breath-hold imaging and treatments.

Deep learning methods have shown promising results in medical imaging processing, such as image classification and reconstruction. Using deep learning methods in synthetic CT generation may be more feasible than voxel-based methods for low-field MRgART to obtain better image quality and may produce more accurate electron/mass density maps for dose calculations. Cusumano et al. (12) demonstrated that using a conditional generative adversarial network (cGAN) was feasible to generate synthetic CT from low-field MR images for abdominal and pelvic cases. Fu et al. (14) compared synthetic CT images generated from 0.35T MR images using the cGAN and the cycle-consistent generative adversarial network (cycleGAN) for liver radiotherapy and reported that both cGAN and cycleGAN achieved accurate dose calculations. Olberg et al. (13) proposed a deep spatial pyramid convolution framework to generate synthetic CT from 0.35T MRI for breast radiotherapy and demonstrated improved performance compared to a conventional GAN framework.

Based on previous studies, GAN-based deep learning methods have the potential to generate high quality synthetic CT images from low-field MR images. However, most of these studies trained networks using a single two-dimensional (2D) plane approach, typically using axial slices. Therefore, discontinuities across slices were often observed with this single 2D plane method. Using three-dimensional (3D) volumes as an input may generate a more accurate synthetic CT volume; however, this approach may require larger datasets for training to obtain high quality results. The aim of the study was to evaluate the quality of synthetic CT images generated by a cGAN using a multi-planar method as inputs in the application of low-field MRgART for prostate radiotherapy.

## 2 Materials and methods

### 2.1 Patient selection and image data

Fifty-seven male patients, who received MRI-guided pelvic radiation therapy on a ViewRay 0.35T MRIdian Linac (ViewRay Inc., Oakwood, OH, USA) between 2019 and 2022, were retrospectively enrolled in this study. All patients were scanned and treated with a free breathing technique. The selection criteria included absence of hip implants and less imaging artifacts, determined by visual interpretation. Each patient had both CT-sim and 0.35T MR-sim images acquired on the same day and in the treatment position. The CT images were acquired on a Siemens SOMATOM Confidence CT simulator (Siemens Healthcare, Malvern, PA, USA) or GE LightSpeed RT16 (GE Healthcare, Chicago, Illinois, USA), with a tube voltage of 120 kVp, slice thicknesses of 2 to 3 mm and in-plane pixel sizes of 0.98 × 0.98 and 1.27 × 1.27 mm^2^ (**Table 1**). MR images were acquired using a TrueFISP sequence on the 0.35T MRIdian, with two different fields of view (**Table 1**). All patients were simulated on both the CT simulator and the MR-linac with a modest bladder filling and enema rectum preparation (3).

**Table 1:** CT imaging parameters (simulator model and voxel size), MR imaging parameters (field of view, scan time, voxel size, flip angle, repetition time and echo time), treatment sites and clinical plan information for training and validation/testing datasets.

**Training Datasets (45)**	CT Parameters	SOMATOM – 0.98×0.98×3 mm^3^ (38)
		SOMATOM – 1.27×1.27×2 mm^3^ (1)
		SOMATOM – 1.27×1.27×3 mm^3^ (4)
		LightSpeed – 0.98×0.98×2.5 mm^3^ (2)
	MRI Parameters	50×45×43 cm^3^– 172 sec (41)
		1.5×1.5×1.5 mm^3^/60°/3.37 ms/1.45 ms
		50×45×43 cm^3^– 25 sec (3)
		1.5×1.5×3.0 mm^3^/60°/3 ms/1.27 ms
		50×30×36 cm^3^– 173 sec (1)
		1.5×1.5×1.5 mm^3^/60°/3.37 ms/1.45 ms
	Treatment Sites	Pelvic Nodes (23)
		Prostate (19)
		Perirectal (1)
		Sacrum (1)
		Ureter (1)
**Validation/Testing Datasets (12)**	CT Parameters	SOMATOM – 0.98×0.98×3 mm^3^ (10)
		SOMATOM – 1.27×1.27×3 mm^3^ (1)
		LightSpeed – 0.98×0.98×2.5 mm^3^ (1)
	MRI Parameters	50×45×43 cm^3^– 172 sec (12)
		1.5×1.5×1.5 mm^3^/60°/3.37 ms/1.45 ms
	Fractionation	36.25 Gy in 5 fractions (8)
		SIB (36.25, 45 Gy) in 5 fractions (4)
	Number of Beams	24 – 29 (avoid posterior beams and entering through couch edges)
	Number of Monitor Units	1795 – 3474 MUs per fraction

Number of patients are indicated in parentheses. SIB, simultaneous integrated boost.

A subset of forty-five cases were used for model training. The treatment sites included pelvic nodes, prostate, perirectal area, sacrum and ureter (**Table 1**). The task for the model was to learn male pelvic anatomy and CT-to-MRI mapping for a large patient cohort. Thus, the training cases were not limited to only prostate cases. The remaining twelve cases were used for model validation/testing. These validation/testing datasets were limited to prostate stereotactic body radiotherapy (SBRT) patients. The treatment plan parameters of these prostate cases are shown in **Table 1**.

### 2.2 Synthetic CT generation

#### 2.2.1 Image preprocessing

In our current clinical workflow, the CT images were deformably registered to 0.35T MR images using MIM (MIM Software Inc., Cleveland, OH, USA) to get electron/mass density information for dose calculations. The quality of deformed CT images was carefully reviewed in the planning process. For our study, the deformed CT and MR images were exported, and additional image preprocessing was performed.

All MR images were corrected for residual intensity non-uniformity using a commonly applied post-processing bias-field correction algorithm (N4itk) (15) and implemented in a publicly available image analysis software environment (SLICER, surgical processing laboratory, Brigham and Women’s Hospital, Boston, MA, USA). The bias field was estimated within the volume defined by the skin surface. The N4itk optimization parameters included: BSpline order of 3, BSpline grid resolutions of (1, 1, 1), a shrink factor of 4, maximum numbers of 50, 40 and 30 iterations at each of the 3 resolution levels, and a convergence threshold of 0.0001. All CT and MR images were resampled to 256×256×256 with a resolution of 1.5×1.5×1 mm^3^. The pixel value of the volume outside the skin surface was set to -1000 for CT images and 0 for MR images.

The pixel value of CT images ranged from -1000 to 3095 while the pixel value for MR images ranged from 0 to a varying maximum value. The CT image intensity was normalized by dividing 4095 and scaled such that the CT number of -1000 HU was mapped to -1 and the CT number of 3095 HU was mapped to 1. The MR intensity was also normalized and mapped from -1 to 1 using a z-score method (16). The z-score method standardizes the image by centering the intensity histogram at a mean of 0 and dividing the intensity by the standard deviation. When calculating the mean and standard deviation for each MRI dataset, the areas with an intensity smaller than 40 were excluded from the calculations. This threshold value is often used in our clinical workflow to define the air/gas region on TrueFISP MR images. After normalizing the MR intensities, any intensity value less than or greater than three standard deviations from the mean were truncated to be -1 and 1, respectively. For example, after normalization, the pixel value in the air region was -1 and the value in the brightest area on MR images was 1.

#### 2.2.2 Deep learning network and loss function

**Figure 1:**
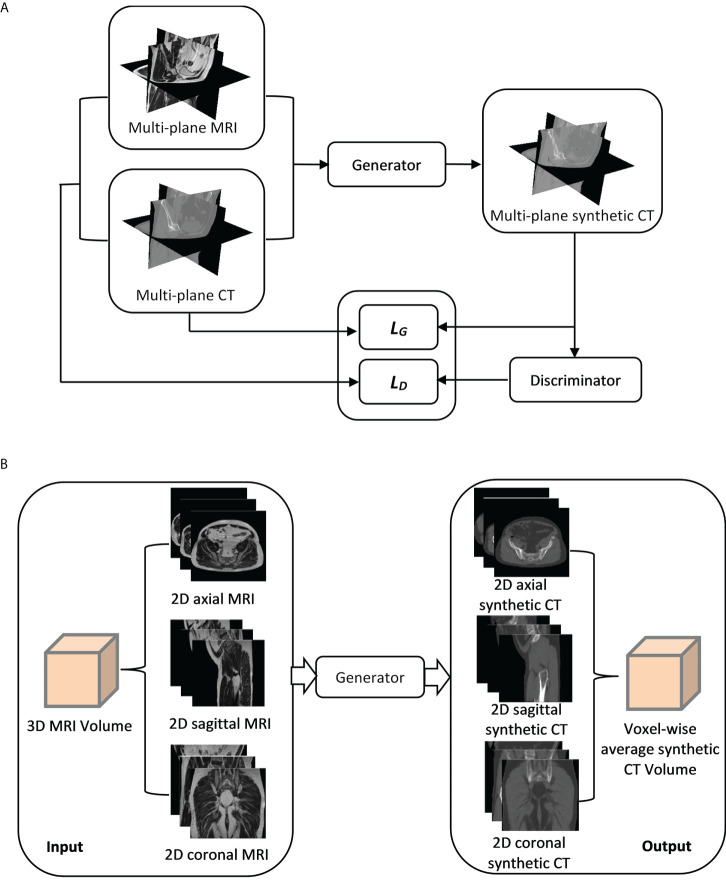
**(A)** A synthetic CT training process using a multi-planar method. Three orthogonal planes from paired MR-CT image sets are used to train generator and discriminator networks with loss functions (*L_G_* and *L_D_*). **(B)** A synthetic CT generation process for validation/testing. A 3D MRI volume is sampled in three orthogonal directions, generating three MRI sets as inputs to the generator. Three corresponding synthetic CT sets are generated (sCT_ax_, sCT_cor_, sCT_sag_) and combined to get the final synthetic CT (sCT_ave_).


A cGAN based on the pix2pix architecture with some modifications was employed to generate synthetic CT images from MR images (17). The GAN has two neural networks, a generator and a discriminator, contesting with each other (**Figure 1A**). The generator (*G*) learns to generate synthetic CT images while the discriminator (*D*) learns to distinguish the generator’s synthetic CT images from real CT images. The generator was based on U-Net architecture with skip-connections while the discriminator was based on 16×16 PatchGAN. The objective (*L_T_*) was composed of an adversarial loss (*L_cGAN_*), pixel reconstruction loss (*L_L1_*) with a weighting factor (λ_1_) and mutual information loss (*L_MI_*) with a weighting factor (λ_2_).

$$L_{T}=arg\underset{G}{\min}\underset{D}{\max}L_{cGAN}\left(G,D\right)+\lambda_{1}L_{L1}\left(G\right)+\lambda_{2}L_{MI}\left(G\right)$$

$$L_{cGAN}(G,D)=E_{x,y}\left[D{\left(x,y\right)}^{2}]+E_{x}[{\left(D(x,G(x))-1\right)}^{2}\right]$$

$$L_{L1}(G)=E_{x,y}[\mathrm{||}y-G{(x)\mathrm{||}}_{1}]=\frac{1}{n}\sum_{i=1}^{n}\left|y_{i}-G(x_{i})\right|$$

$$L_{MI}(G)=E_{x,y}\left[1-nMI(y,G(x))\right]$$

where λ_1_ and λ_2_ were 100 and 5, respectively, and *x*, *y* and *G*(*x*) represent MR images, real CT images and generated synthetic CT images, respectively. For *L_cGAN_*, a least-squares objective was used rather than the log likelihood objective because former exhibited better stability during training (18). The *L_L1_* objective was used to calculate the mean-absolute-error between real CT and generated synthetic CT images for all voxels (*n*). The *L_MI_* objective was not in the original pix2pix implementation but was added in the loss function to minimize the effect of the uncertainty due to the misalignment between CT and MR images. This objective was to calculate image similarity between real CT and synthetic CT images, and the mutual information was normalized (*nMI*) so it ranged from 0 to 1.

To reduce the model oscillation, the discriminator was updated using a history of generated images rather than the ones produced by the latest generator (18). The optimization was performed using an adaptive moment estimation (Adam) solver, with an initial learning rate of 0.0002 for the generator and 0.00005 for the discriminator and momentum parameters of 0.5 and 0.999 (17). The learning rate was decreased by 1% per increasing epoch. A total of 100 epochs were trained.

The network training was performed in MATLAB (MathWorks, Inc., Natick, MA, USA) using a RTX A5000 GPU (NVIDIA, Sunnyvale, CA, USA). The GAN was trained on three orthogonal directions. Each image batch in the iteration of the training process included 2D axial, sagittal and coronal planes of the paired MR and CT images (**Figure 1A**). In each iteration, three orthogonal planes were randomly extracted from 3D volumes (unused slices in prior iterations) in both MR and CT image sets. If no anatomy information existed in any extracted planes, that image batch was disregarded. Maximum iterations in each epoch were 256 (slices)×45(number of training cases).

#### 2.2.3 Synthetic CT generation for validation/testing

The generator network was trained with the multi-planar method (**Figure 1A**). Thus, an MRI volume sampled in any orthogonal direction can be used as an input to generate a synthetic CT volume. To improve the robustness of synthetic CT generation and reduce discontinuities across slices, three image sets sampled in three orthogonal directions from each MRI volume were input into the generator and the corresponding synthetic CT image sets in 2D axial (sCT_ax_), coronal (sCT_cor_) and sagittal (sCT_sag_) planes were created, as illustrated in **Figure 1B**. As a result, three predictions were acquired for each voxel. Predicted values from the outputs in all three directions were then averaged to obtain the final synthetic CT volume (sCT_ave_). The synthetic CT generation was performed using an in-house program, written in MATLAB.

### 2.3 Evaluation of synthetic CT quality

#### 2.3.1 Image quality of synthetic CT volumes

To evaluate the quality of synthetic CT images, their similarity to a ground truth CT, on a voxel-wise basis, was calculated. The CT images closest to the ground truth in this study were deformed CT images. Therefore, mean-absolute-errors (MAE) and mean errors (ME) in CT numbers, peak signal-to-noise ratios (PSNRs) and structural similarity index measures (SSIMs) between synthetic CT and deformed CT were calculated following the equations shown below.


$$MAE=\frac{\sum_{1}^{n}\left|sCT_{i}-CT_{i}\right|}{n}$$

$$ME=\frac{\sum_{1}^{n}sCT_{i}-CT_{i}}{n}$$

$$PSNR=10\text{log(}\frac{Max_{CT}^{2}}{MSE})$$

$$SSIM=\frac{\left(2\mu_{sCT}\mu_{CT}+c_{1})(2\sigma_{sCT,CT}+c_{2}\right)}{\left(\mu_{sCT}^{2}+\mu_{CT}^{2}+c_{1})(\sigma_{sCT}^{2}+\sigma_{CT}^{2}+c_{2}\right)}$$

where *n* is the voxel number in the region of interest (ROI); *MAX_CT_* is the maximum pixel value of deformed CT images; *MSE* is the mean-squared-error; μ_sCT_ and μ_CT_ are the means of synthetic CT and deformed CT images;  σ_sCT_ and σ_CT_ are the standard deviations;  σ_sCT, CT_ is cross-covariance; c_1_ and c_2_ are regularization constants for the luminance and contrast.

The ROI in calculating the SSIM was the whole 3D volume (256×256×256), while the ROI in calculating the PSNR, MAE and ME was the 3D volume but only included the voxels within the body contour. In particular, the MAE and ME were also calculated in three different tissue segments, including air, soft tissue and bone. The three tissue segments were identified by applying image intensity thresholds on the deformed CT. The voxels with CT numbers smaller than -200 HU was included in the air segment, and the voxels with CT numbers larger than 200 HU was included in the bone segment. The residual voxels were included in the soft tissue segment, i.e. CT numbers from -200 HU to 200 HU. No morphological post-processing on these segments was performed.

#### 2.3.2 Dosimetric accuracy of synthetic CT volumes

Clinical intensity-modulated radiotherapy plans were calculated on deformed CT images. As the deformation was not likely perfect, differences in body contours and gas pockets between MRI and deformed CT datasets were compensated by overriding the mass density (relative to water) in these structures with soft tissue (1.02) and air (0.0012). Dose distributions were calculated using a Monte Carlo algorithm implemented in the MRIdian treatment planning system, including the effect of the magnetic field, 0.2 cm calculation grid and 0.5% calculation uncertainty. The clinical plans were then recalculated on the synthetic CT images with the same fluence and calculation parameters for each validation case. The CT calibration curve used in the synthetic CT recalculation was the same as the one in the initial plan, depending on which simulator was used for CT image acquisition.

Dose volume histograms (DVHs) were compared between synthetic CT and user-corrected deformed CT (dCT_corr_) calculations of each clinical plan. Two DVH metrics (D95% and D2%) were evaluated for planning target volumes (PTVs) and D2% was evaluated for organs-at-risk (OARs), including rectum, bladder and urethra, for prostate SBRT.

Three-dimensional dose distributions were exported to SLICER and 3D gamma analyses were performed in a ROI where the dose was larger than 10% of the maximum dose in each clinical plan. Two different criteria in gamma analyses were compared, 1%/1 mm and 2%/2 mm.

## 3 Results

### 3.1 Image quality of synthetic CT volumes

**Figure 2:**
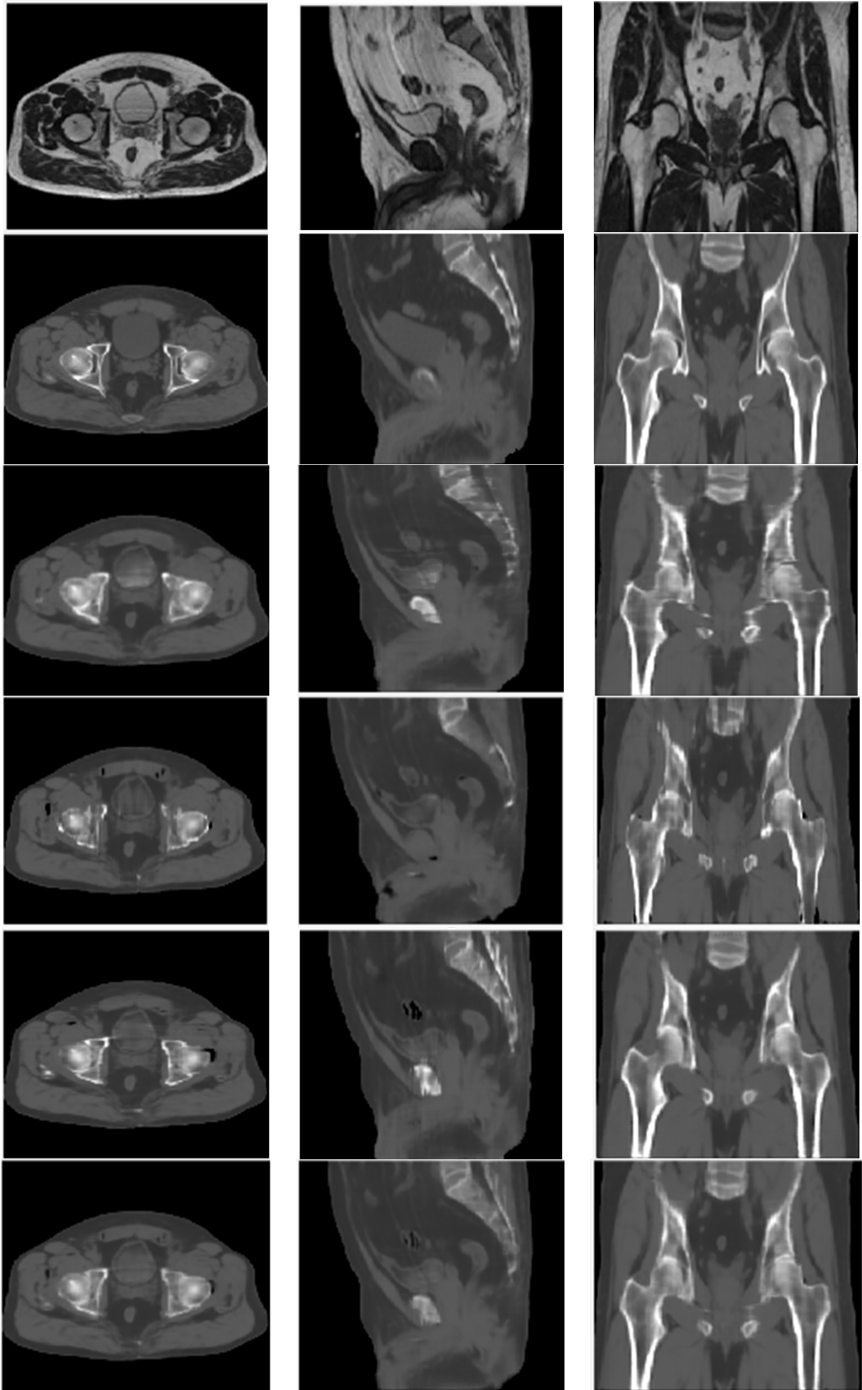
MR (top row), deformed CT (2^nd^ row), sCT_ax_ (3^rd^ row), sCT_sag_ (4^th^ row), sCT_cor_ (5^th^ row) and sCT_ave_ (bottom row) for one of the validation cases.

The image quality of all synthetic CT image sets generated along different orientations was similar to the quality of deformed CT image sets (**Figure 2**). However, for sCT_ax_ image sets, the axial view had smoother edges than the sagittal and coronal views. Similar behaviors were observed for sCT_sag_ and sCT_cor_ image sets. The sCT_ave_ image set was created by averaging sCT_ax_, sCT_sag_ and sCT_cor_ image sets, so the discontinuities across slices, observed in sCT_ax_, sCT_sag_ and sCT_cor_ image sets, were reduced.

Regarding quantitative analyses, the MAEs ranged from 30.1 HU to 33.6 HU in the whole pelvis area for all four synthetic CT image sets; the MEs ranged from -4.3 HU to -7.9 HU; PSNRs ranged from 33.8 to 35.2; SSIMs ranged from 0.9694 to 0.9758 (**Table 2**). In terms of CT numbers in various tissue segments, the accuracy was the best in the soft tissue segment and worst in the air segment, due to changes in bowel gas positioning between scans. Comparing sCT_ave_ with the other three synthetic CT sets, MAEs were ~10% lower, PSNRs were 3% higher, and SSIMs were 0.5% higher in the whole pelvis.

**Table 2:** MAEs and MEs for whole pelvis and individual segments (air, soft tissue and bone) and PSNRs and SSIMs for whole pelvis.

	Axial (sCT_ax_)	Sagittal (sCT_sag_)	Coronal (sCT_cor_)	Average (sCT_ave_)
Whole pelvis (MAE)	32.8 ± 4.4 [26.4 to 39.1]	33.6 ± 4.4 [27.7 to 40.5]	33.2 ± 4.4 [26.2 to 38.5]	30.1 ± 4.2 [23.7 to 36.1]
Air (MAE)	406.2 ± 119.3 [176.3 to 558.6]	402 ± 131.6 [160.2 to 539.5]	409.8 ± 113.7 [207.0 to 564.2]	396.5 ± 126.1 [159.3 to 542.1]
Soft tissue (MAE)	21.6 ± 2.3 [19.2 to 25.4]	21.4 ± 2.6 [18.9 to 25.8]	21.8 ± 2.9 [18.9 to 27.9]	19.6 ± 2.3 [17.4 to 23.6]
Bone (MAE)	171.7 ± 25.8 [126.4 to 216.5]	188.7 ± 25.6 [140.5 to 243.1]	175.9 ± 29.9 [121.5 to 215.0]	158.5 ± 26.0 [111.9 to 209.7]
Whole pelvis (ME)	-4.3 ± 4.4 [-11.1 to 2.2]	-7.5 ± 4.0 [-16.1 to -1.6]	-7.9 ± 4.2 [-14.8 to -2.9]	-6.8 ± 3.6 [-14.3 to -2.8]
Air (ME)	395.7 ± 122.9 [165.7 to 558.3]	381.0 ± 146.7 [132.2 to 535.4]	394.8 ± 120.1 [191.1 to 551.3]	388.4 ± 131.3 [148.9 to 541.2]
Soft tissue (ME)	0.7 ± 3.3 [-6.4 to 5.1]	-1.1 ± 2.8 [-7.3 to 4.0]	-2.5 ± 2.4 [-5.6 to 1.6]	-1.2 ± 2.4 [-6.4 to 3.1]
Bone (ME)	-115.9 ± 40.9 [-184.6 to -58.2]	-142.5 ± 34.9 [-221.7 to -81.0]	-125.6 ± 43.7 [-189.2 to -41.6]	-128.0 ± 35.2 [-198.5 to -65.4]
PSNR	34.4 ± 1.5 [31.9 to 36.8]	33.8 ± 1.4 [31.2 to 35.9]	34.0 ± 1.4 [31.9 to 36.3]	35.2 ± 1.7 [32.4 to 38.0]
SSIM	0.9722 ± 0.0031 [0.9669 to 0.9765]	0.9706 ± 0.0047 [0.9634 to 0.9771]	0.9694 ± 0.0053 [0.9576 to 0.9752]	0.9758 ± 0.0035 [0.9705 to 0.9800]

Means, standard deviations and ranges are shown in the table.

### 3.2 Dosimetric accuracy of synthetic CT volumes

**Figure 3:**
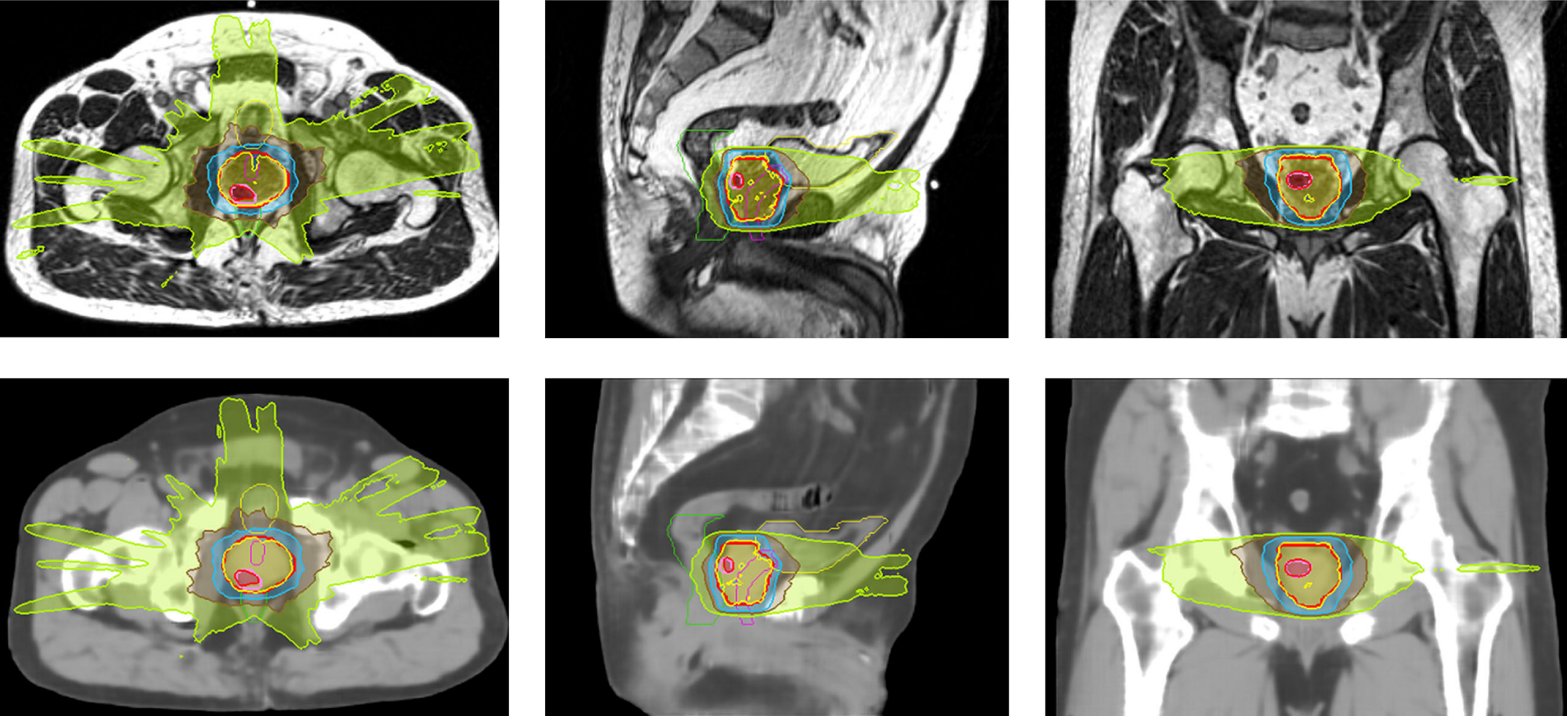
Isodose color wash (124%, 100%, 69%, 50% and 30% of 36.25 Gy in the prescribed dose) displayed on MR and sCT_ave_ images in axial, sagittal and coronal views for the best case (SIB case).

**Figure 4:**
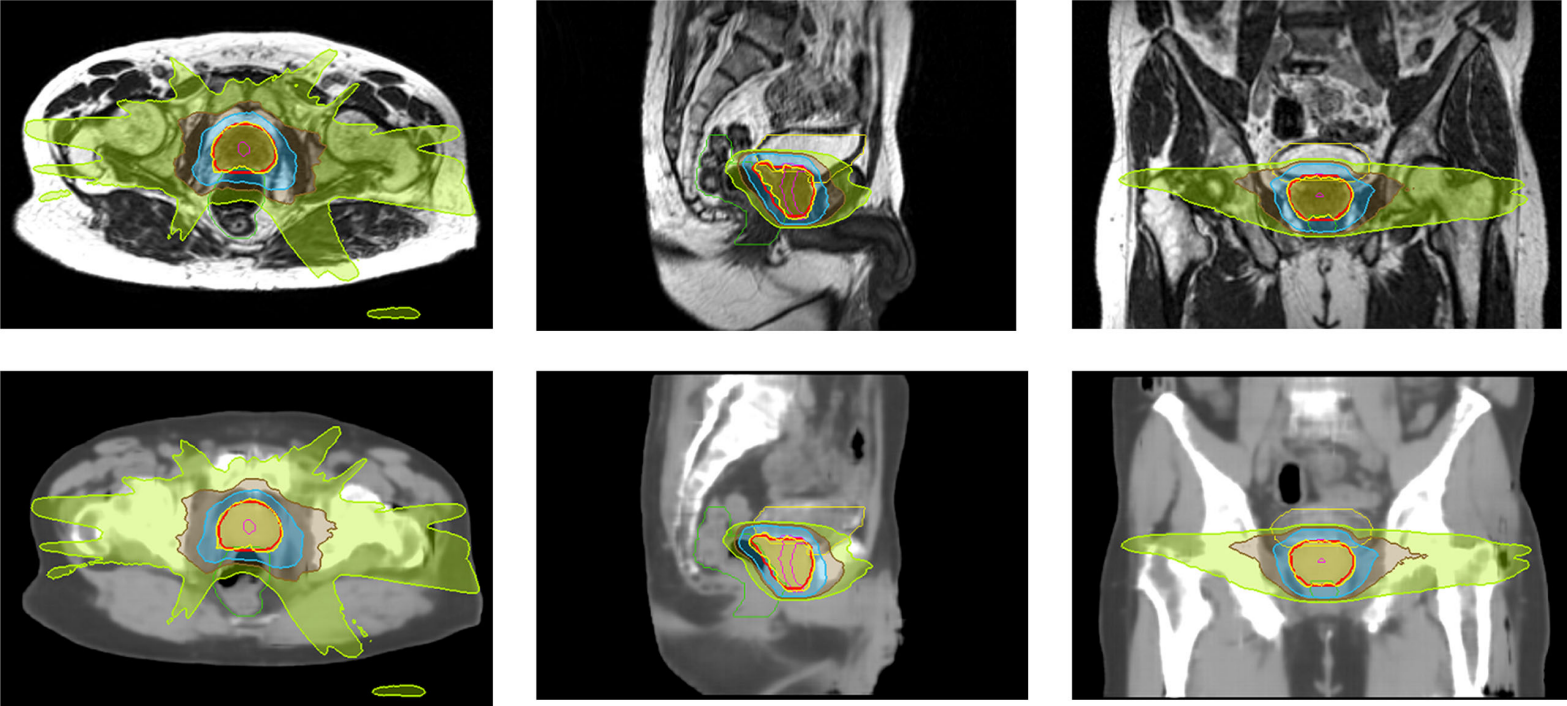
Isodose color wash (100%, 69%, 50% and 30% of 36.25 Gy in the prescribed dose) displayed on MR and sCT_ave_ images in axial, sagittal and coronal views for the worst case.

**Figure 5:**
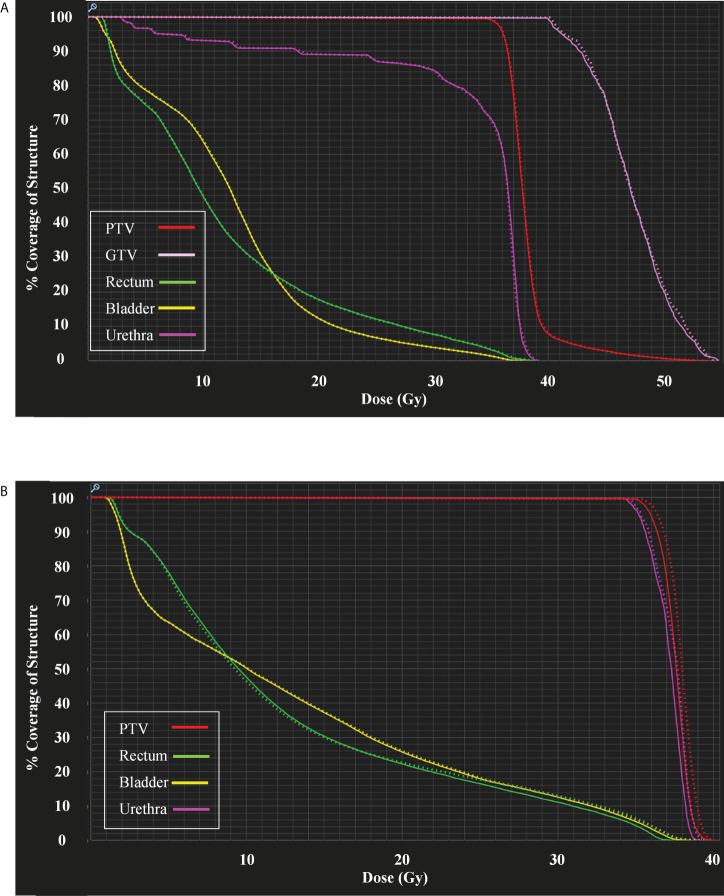
**(A)** DVHs for the case shown in **Figure 3** and **(B)** DVHs for the case shown in **Figure 4**. Sold lines: dCT_corr_; dotted lines: sCT_ave_.

Clinical plans were recalculated on sCT_ave_ image sets. **Figure 3** plots the case that exhibited the best agreement in CT numbers (23.7 HU in MAE for the whole pelvis) and DVH metrics (< 0.2 Gy), while **Figure 4** shows the case that had the largest differences in CT numbers (36.1 HU in MAE for the whole pelvis) and DVH metrics (< 0.9 Gy). The DVHs for the best and worst cases are shown in **Figure 5**. Large dose differences were observed in the PTV, urethra and rectum for the worst case.

**Figure 6:**
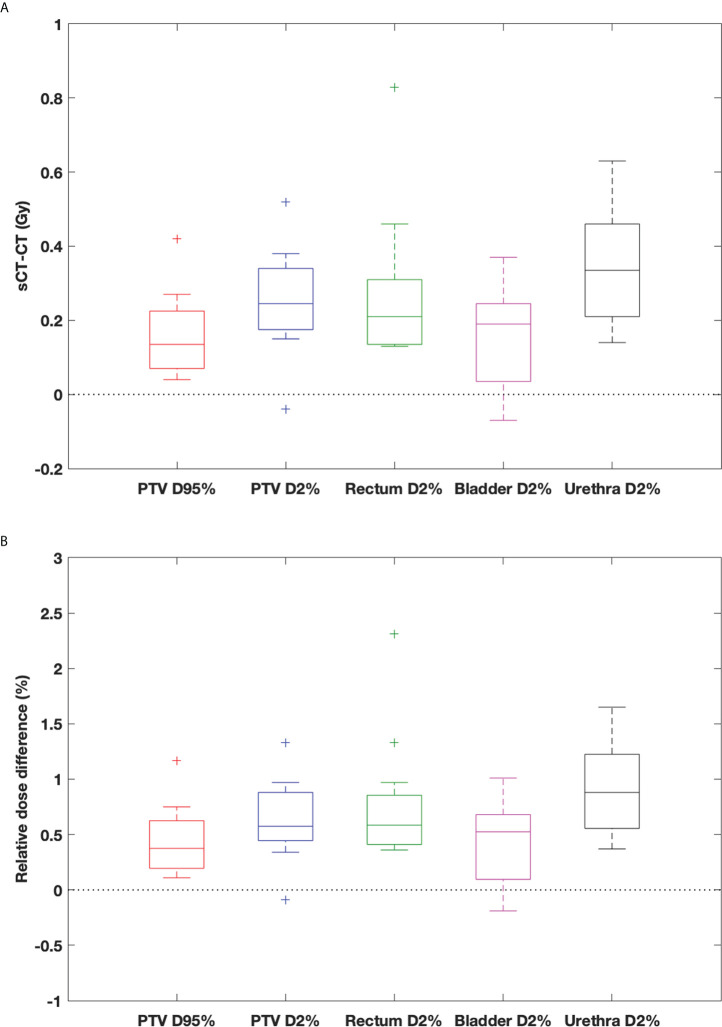
Box-and-whisker plots of **(A)** dose difference (Gy) and **(B)** relative dose difference (%) between sCT_ave_ and dCT_corr_ for PTV and OAR metrics. The bottom and top of the box represent the 1st and 3rd quartiles; the band inside the box is the median; the ends of the whiskers represent 95% range; the crosses represent outliers.

In general, the DVH metrics calculated on sCT_ave_ image sets were higher than those calculated on dCT_corr_ image sets (**Figure 6** and **Table 3**). The mean dose differences were within 0.25 Gy for the PTV metrics and 0.35 Gy for the OAR metrics, representing 0.6% and 0.9%, respectively (**Table 3**). All validation cases had dose differences less than 0.7 Gy in both PTV and OAR structures except one case (the worst case shown in **Figure 4**) that had a deviation in rectal D2% of 0.9 Gy (2.3%) (**Figure 6**).

**Table 3:** DVH metrics of dCT_corr_ and sCT_ave_, the comparison between sCT_ave_ and dCT_corr_ in DVH metrics and 3D gamma analyses with 1%/1 mm and 2%/2 mm in a ROI where the dose is larger than 10% of the maximum dose in each clinical plan.

	dCT_corr_ (Gy)	sCT_ave_ (Gy)	sCT_ave_ vs dCT_corr_ (Gy)	sCT_ave_ vs dCT_corr_ (Relative)	3D Gamma	
					1%/1mm	2%/2mm
PTV D95%	36.11 ± 0.18	36.27 ± 0.16	0.16 ± 0.11	0.5% ± 0.3%	99.0% ± 0.8%	99.9% ± 0.1%
PTV D2%	40.83 ± 3.26	41.09 ± 3.18	0.25 ± 0.14	0.6% ± 0.4%		
Rectum D2%	35.53 ± 1.91	35.80 ± 1.94	0.27 ± 0.20	0.8% ± 0.6%		
Bladder D2%	35.84 ± 0.75	35.98 ± 0.79	0.15 ± 0.14	0.4% ± 0.4%		
Urethra D2%	37.95 ± 0.33	38.30 ± 0.33	0.35 ± 0.15	0.9% ± 0.4%		

Comparing dose distributions using 3D gamma analyses, good agreement was found for all validation cases, with 99% and 99.9% pass rates for 1%/1 mm and 2%/2 mm criteria, respectively (**Table 3**).

## 4 Discussion

The multi-planar cGAN deep learning method used in this study to generate synthetic CT images reduced discontinuities across slices that were often observed in the 2D planar method. The MAE in CT numbers was reduced by ~10% with the multi-planar method compared to the 2D planar method. Regarding its dosimetric accuracy compared to clinical plans on dCT_corr_, the mean relative difference was less than 1% for PTV and OAR metrics, and 3D gamma analyses showed good agreement, achieving a 99% pass rate for 1%/1 mm criteria. This study demonstrated that the presented method generated synthetic CT images comparable to the deformed CT method that is currently used in the low-field MRgART workflow for treating prostate cancer.

Some outliers were observed when evaluating the dosimetric accuracy, particularly in the rectum (**Figures 4**, **5B**, **6**). Several factors may contribute to the large deviations. First, the MRIdian TPS uses a Monte Carlo method, which has an inherent calculation uncertainty (0.5% in this study). Second, deformed CT images with air and soft tissue overrides were used as ground truth to validate the accuracy of synthetic CT images. However, anatomical changes may be observed between simulation CT and MR scans, despite their acquisition on the same day, e.g., changes in bladder filling (as seen on the MR and CT images in **Figure 2**) and variation of gas pockets in the bowel and rectum (resulting in large deviations for air segments in **Table 2**). To compensate for the change in gas morphology, manual density overrides with pure air were used when planning. This process can introduce additional uncertainties because gas pockets may not be correctly identified nor necessarily be composed of pure air. As shown in **Figure 4**, a large gas pocket was found in the rectum adjacent to the prostate. The accuracy of its size and density was crucial for this case because of the electron return effect in the presence of the magnetic field. It is therefore difficult to assert that the deformed CT with density overrides represents the ground truth. Ultimately this resulted in higher measured error.

Compared with previous synthetic CT generating studies in the prostate or pelvis using deep learning methods, our results were superior or comparable to those performed with either low-field (12, 19) or high-field (14, 20–25) MRI. For example, Cusumano et al. (12) generated synthetic CT from 0.35T MRI using a 2D cGAN and reported that MAEs in CT numbers were 54.3 ± 11.9 HU, 40.4 ± 9.2 HU, 224.4 ± 35.5 HU for whole pelvis, soft tissue and bone, respectively, as compared to our results, 30.1 ± 4.2 HU, 19.6 ± 2.3 HU and 158.5 ± 26.0 HU for whole pelvis, soft tissue and bone, respectively. Fu et al. (14) used T1-weighted images from 1.5T MRI to synthesize CT using a 3D convolutional neural network (CNN) and reported 37.6 ± 5.1 HU, 26.2 ± 4.5 HU and 154.3 ± 22.3 HU for whole pelvis, soft tissue and bone, respectively. Chen et al. (22) used T2-weighted images from 3T MRI to synthesize CT using a 2D U-net model and reported 30.0 ± 4.9 HU, 19.6 ± 2.5 HU and 122.5 ± 10.5 HU for whole pelvis, soft tissue and bone, respectively.

Regarding the calculation time, the time required by the neural network to generate synthetic CT was ~12 seconds with the GPU system used in this study. The presented method would be more efficient and provide more accurate electron/mass density information as the density override and review processes take ~4 minutes in our current MRgART workflow.

The image resolution and size combination used in this study may cut off part of the anatomy for fields of view larger than 38.4 cm×38.4 cm and limit potential clinical implementation in larger patients. A follow-up study will consider increasing the image size to ensure the method can be applied to large-size patients.

In addition, future work will investigate the accuracy, robustness, and limitations of the presented method in a large patient cohort, apply the method to different body sites for MRgART and explore a method to independently verify the accuracy of the synthetic CT generation for a quality assurance (QA) process in the clinical workflow, such as catching outliers to trigger further review in MRgART. A QA process will ensure that the deep learning method can be safely and reliably implemented in clinical workflows to generate synthetic CT volumes from MRI volumes and to improve the workflow efficiency in MRI-guided radiotherapy.

## 5 Conclusion

The quality of synthetic CT images generated from 0.35T MRI using a multi-planar cGAN method was evaluated for prostate radiotherapy. The MAE was 30.1 ± 4.2 HU in the whole pelvis compared to the deformed CT. Calculated doses on synthetic CT images agreed well with the doses in the clinical plans with a gamma pass rate of 99% for 1%/1 mm criteria, and the difference was less than 1% on average for all PTV and OAR metrics. Our study demonstrates the potential of using synthetic CT created from 0.35T MRI TrueFISP images in the adaptive workflow of prostate radiotherapy. Future work will validate the method in a large cohort of patients and investigate the limitations of the method in the adaptive workflow.

## Data availability statement

The original contributions presented in the study are included in the article/supplementary material. Further inquiries can be directed to the corresponding author/s.

## Ethics statement

The studies involving human participants were reviewed and approved by IRB - 2020P000292: Retrospective Data Analysis of Patients Treated on the MR-Linac. Written informed consent for participation was not required for this study in accordance with the national legislation and the institutional requirements.

## Author contributions

SH designed the study, developed the method, analyzed data and drafted the manuscript. All authors contributed to the article and approved the submitted version.

## Acknowledgments

AS’ effort on this project was supported by the NIBIB of the National Institutes of Health under award number R21EB026086. The content is solely the responsibility of the authors and does not necessarily represent the official views of the National Institutes of Health.

## Conflict of interest

JL has received research funding from ViewRay and NH TherAguix.

The remaining authors declare that the research was conducted in the absence of any commercial or financial relationships that could be construed as a potential conflict of interest.

## Publisher’s note

All claims expressed in this article are solely those of the authors and do not necessarily represent those of their affiliated organizations, or those of the publisher, the editors and the reviewers. Any product that may be evaluated in this article, or claim that may be made by its manufacturer, is not guaranteed or endorsed by the publisher.
